# Initial attitudes toward a drug predict medication adherence in first-episode patients with schizophrenia: a 1-year prospective study in China

**DOI:** 10.1186/s12888-023-05419-y

**Published:** 2023-12-05

**Authors:** Nan Dai, Bingjie Huang, Tianqi Gao, Yue Zheng, Chuan Shi, Chengcheng Pu, Xin Yu

**Affiliations:** 1 Department of Psychiatry, First Affiliated Hospital of Kunming Medical University, 295 Xichang Road, Kunming, Yunnan China; 2 Peking University Sixth Hospital, Peking University Institute of Mental Health, NHC Key Laboratory of Mental Health (Peking University), National Clinical Research Center for Mental Disorders (Peking University Sixth Hospital), 51 Huayuan North Road, Beijing, China

**Keywords:** Schizophrenia, Patients compliance, The Drug Attitude Inventory

## Abstract

**Background:**

Patients’ attitudes toward medication have been shown to be a predictor of nonadherence to antipsychotic treatment. However, most previous studies that explored this relationship used a cross-sectional design. It is important to explore the association of attitudes toward drugs with discontinuation at different time points during antipsychotic treatment. In this study, we investigated the association of attitudes toward drugs (measured by the Drug Attitude Inventory (DAI-10)) with adherence at seven time points (baseline, 4 weeks, 8 weeks, 12 weeks, 26 weeks, 39 weeks, and 52 weeks) during 1 year of treatment. Factors that were potentially associated with attitudes toward drugs at the time point of interest were also studied.

**Methods:**

Demographic characteristics, psychopathology, social functioning, and attitudes toward drugs (measured by the DAI-10) were collected at baseline, 4 weeks, 8 weeks, 12 weeks, 26 weeks, 39 weeks and 52 weeks. The association of attitudes toward drugs (measured by DAI-10) with adherence at the seven time points was calculated using the Mann‒Whitney U test. The optimal cutoff point for the DAI-10 was then determined using receiver operating characteristic (ROC) analysis. Cox regression analysis was conducted to further investigate the association of DAI-10 scores with discontinuation, controlling for potential confounding variables. We used multiple regression analysis to identify the factors associated with DAI-10 scores.

**Results:**

Among the six time points, only baseline DAI-10 total scores were significantly different between the completed and discontinued groups (*p* = 0.004). Female sex and a baseline DAI-10 total score greater than − 1 were found to be independent protective factors against discontinuation of antipsychotic drug treatments during the 1-year follow-up. At baseline, the severity of the disease (CGI-s) and insight regarding the disease were shown to be associated with DAI-10 total scores.

**Conclusion:**

Attitudes toward antipsychotic drugs at baseline were shown to play a crucial role in predicting treatment discontinuation.

**Trial registration:**

The data were collected from a clinical trial and the clinical trials.gov ID of the study is NCT01057849.

## Introduction

Schizophrenia is a chronic psychotic disease with a lifetime prevalence of 1%. Antipsychotic medications are the first-line treatment for patients with schizophrenia, and all international treatment guidelines recommend the long-term use of antipsychotic drugs for schizophrenia. Nevertheless, only half of patients with schizophrenia adhere to their antipsychotic medication regimens [1, 2]. Nonadherence to medication among patients with schizophrenia has been shown to be associated with a higher risk of relapse and rehospitalization [3]. In most studies, nonadherence has even been shown to be one of the most important risk factors for relapse, rehospitalization, treatment resistance, suicide attempts, and impaired long-term functioning in patients with schizophrenia [4]. Nonadherence to medication has thus become a critical issue in the management of schizophrenia.

Recently, attention has been devoted to identifying predictors of medication discontinuation or nonadherence in first-episode [5, 6] or multiple-episode patients with schizophrenia. Previous studies have shown that one of the strongest predictors of nonadherence is patients’ attitudes toward medication [6, 7]. Attitudes toward drug adherence may reflect subjective attitudes about the side effects of medication, long-term antipsychotic treatments and schizophrenia itself.

The Drug Attitude Inventory (DAI) is the most widely used instrument to evaluate attitudes toward drugs among patients with schizophrenia [8]. The DAI is a self-report scale with two versions: the DAI-30 and the DAI-10. Compared to the DAI-30, which is composed of 30 items, the brief 10-item version (DAI-10) is easier for patients to complete and easier for clinicians to administer. Additionally, the psychometric properties of the DAI-10 have been shown to be comparable to those of the DAI-30 when assessing patients with schizophrenia [9]. The DAI-10 includes 10 true/false items, and the sum scores range from − 10 (very poor attitude) to 10 (best possible attitude). Positive total scores are interpreted as a positive subjective response, and negative total scores are interpreted as a negative subjective response.

To date, the DAI-10 has been used to investigate the association of attitudes toward medication with nonadherence to medication in patients with schizophrenia [10–12]. Previous studies have found that the DAI is associated with the discontinuation of antipsychotic treatments [6, 11]. However, these previous studies used a cross-sectional design [7, 10, 13]. Exploring the association of DAI-10 scores with drug discontinuation at different time points during a study period would provide additional evidence regarding attitudes toward drugs at multiple stages of treatment. Additionally, different cultural backgrounds may have an influence on attitudes toward medication in patients with schizophrenia [14]. Nonetheless, attitudes toward antipsychotic medication have not been well studied in Chinese individuals.

Although various clinical, sociodemographic and social factors can potentially affect medication adherence in patients with schizophrenia [15], dominant influencing factors may vary by country. Several cross-sectional studies conducted with schizophrenia patients in China suggested influencing factors related to medication adherence. Nevertheless, the results in the different studies were inconsistent. For instance, in patients affected by schizophrenia with an episodic course, sociodemographic (age and income) and clinical (being in the acute period of the disease and severity of illness) characteristics were reported to have an impact on medication adherence [16]. For Chinese hospitalized persons with schizophrenia, neither sociodemographic (sex, marital status, and education level) nor clinical characteristics (positive symptoms, negative symptoms and general Psychopathology) were shown to be associated with medication adherence [17].

Attitudes toward antipsychotic medication and their influencing factors may also vary by country. Compared to inpatients with schizophrenia in the United States, inpatients with schizophrenia in China had worse attitudes toward medication (measured by DAI) after controlling for severity of schizophrenia symptoms [18], Moreover, in Chinese outpatients with schizophrenia, female gender, younger age and less severity of illness were related to better attitudes toward medication (measured by DAI). However, longer duration of illness and lower subjective distress caused by side effects predicted better attitudes toward medication in Japanese outpatients with schizophrenia [19].

In this study, data were collected from a large sample of Chinese patients with first-episode schizophrenia, and we carried out a follow-up study to explore the association of attitudes toward drugs (measured by the DAI-10) with medication adherence at six time points (baseline, 4 weeks, 8 weeks, 12 weeks, 26 weeks, and 39 weeks) over 1 year. The potential factors associated with attitudes toward drugs at the time point of interest were also investigated.

In clinical settings, adherence to drug treatment has been a major concern among mental health practitioners, as adherence is usually a key factor in the success of schizophrenia treatment. Our study could provide additional evidence regarding when to evaluate the attitudes toward drugs of patients diagnosed with schizophrenia and which factors could be useful for improving attitudes toward drugs.

## Materials and methods

### Subjects and design

The data were collected from a 1-year randomized, open-labeled clinical trial conducted in 6 major psychiatric hospitals in China. The procedure of this trial has been described elsewhere [20]. Briefly, all of the patients were Chinese and were recruited from three hospitals in the North and three hospitals in the South. All six hospitals were in urban regions, and they provided service to patients from both urban and rural regions. The inclusion criteria for patients were as follows: (1) 18 to 45 years of age; (2) inpatients or outpatients; (3) diagnosis of schizophrenia by the Structured Clinical Interview for the Diagnostic and Statistical Manual of Mental Disorders, Fourth Edition, Text Revision (DSM-IV-TR) Axis I Disorder, patient edition (SCID-I/P); (4) illness duration ≤ 3 years (the onset was determined in the SCID-I/P based on when the first symptom appeared); 5) continuous antipsychotic treatment < 4 weeks and cumulative length of antipsychotic treatment < 12 weeks, with no prior exposure to long-acting antipsychotic injection; and 6) the ability to understand the contents of the interview and provide written informed consent. The exclusion criteria were as follows: (1) current major medical conditions; (2) current or lifetime history of alcohol/drug abuse or dependence; 3) contraindication to olanzapine, aripiprazole or risperidone. All participants or their legal guardians signed the informed consent form after receiving a complete description of the study. The trial was also approved by the ethics committees of the participating hospitals.

Data were collected at baseline, 4 weeks, 8 weeks, 12 weeks, 26 weeks, 39 weeks and 52 weeks. The data included demographic characteristics, psychopathology (the Positive and Negative Syndrome Scale (PANSS) and Clinical Global Impression (CGI)), social functioning (the Personal and Social Performance (PSP) scale), and attitudes toward drugs (the DAI-10). The PANSS was used to assess the presence and severity of mental symptoms. PANSS included 6 items on the positive scale, 6 items on the negative scale and 16 items on the general psychopathology scale [21]. The PSP scale evaluated functioning across four dimensions, including socially useful activities, personal and social relationships, self-care, and disturbing and aggressive behaviors [22]. CGI was used as a brief evaluation of the clinician’s view of the patient’s global functioning prior to and after a study medication. The Clinical Global Impression – Severity scale (CGI-s) used a seven-point scale to rate illness severity at the time of assessment. The psychometric properties of Chinese version of PANSS, PSP and DAI have been reported to be satisfactory [22–24].

Nonadherence or discontinuation was defined as (1) discontinuation of antipsychotics regardless of follow-up completion or (2) early discontinuation of the follow-up (patients who missed follow-ups and then returned were considered as continuation, as long as they remained on the antipsychotic during the missed period).

### Statistical analysis

The patients who completed 52 weeks of treatment and the DAI-10 questionnaire at all 7 investigated time points (baseline, weeks 4, 8, 12, 26, 39 and 52) were defined as the completed group. The patients who discontinued during 52 weeks of follow-up treatments and completed the DAI-10 questionnaire at all investigated time points before discontinuation were identified as the discontinued group.

We first conducted descriptive analyses to examine the DAI-10 total scores at baseline in the discontinued and completed groups. At each investigated time point (baseline, 4 weeks, 8 weeks, 12 weeks, 26 weeks, 39 weeks and 52 weeks), the differences in DAI-10 total scores between discontinued and completed groups were calculated using the Mann‒Whitney U test. Based on the above analysis, baseline DAI-10 total scores were identified as the DAI-10 scores of interest. The DAI scores were then examined with receiver operating characteristic (ROC) analysis to identify the optimal cutoff point.

Based on the selected cutoff score, the role of baseline DAI-10 scores as the predictor of discontinuation was tested using a Cox regression model. The regression analysis needed to control for potentially confounding effects. Therefore, ten indicators that have been reported to have an effect on discontinuation of treatment (age, education, sex, CGI-s, PANSS-Negative scale, PANSS-Positive scale, PANSS-General psychopathology scale, PANSS-G12, PANSS total score, and PSP) were included. Univariate analysis was performed to examine the differences in the 10 confounding variables between the discontinued and completed groups. All indicators that were statistically significant in the univariate analysis were subsequently entered into the Cox regression analysis as control variables. The following parameters were included in the Cox regression model: (1) Status: discontinuation; (2) Time: days (before discontinuation). Finally, a survival curve was constructed.

In the present study, the correlations of baseline DAI-10 total with the 10 confounding variables were also examined. Multiple regression analysis was performed to identify which factors were associated with baseline DAI-10 total scores.

For all statistical tests, a two-tailed *p* < 0.05 was considered statistically significant. The Bonferroni correction was used for multiple testing adjustments. Although the Bonferroni correction should be used for reference, the uncorrected *P* values were also reported. All statistical analyses were performed using SPSS 22.0 (IBM, Armonk, NY, USA) for Windows.

## Results

### Predictors for discontinuation

A total of 430 participants were included in the present study. The 96 patients who completed 52 weeks of treatment and completed the DAI-10 questionnaire at all 7 investigated time points (baseline, 4 weeks, 8 weeks, 12 weeks, 26 weeks, 39 weeks and 52 weeks) were defined as the completed group. A total of 104 patients discontinued during 52 weeks of follow-up treatment and completed the DAI-10 questionnaire at all investigated times before discontinuation; these patients could thus be used to examine the DAI-10 total scores before discontinuation and were identified as the discontinued group.

After we tested the difference in DAI-10 total scores between the completed and discontinued groups at baseline, 4 weeks, 8 weeks, 12 weeks, 26 weeks, and 39 weeks, only the DAI-10 total score at baseline was significantly different (*p* = 0.013). Using ROC analysis, the DAI-10 total score at baseline yielded an AUC = 0.607, which is significantly different from a curve obtained by chance (*p* = 0.014). According to the sensitivity and specificity parameters, the cut off value of the baseline DAI-10 total score was suggested as -1.

Univariate analyses revealed that none of the 10 confounding factors mentioned in the literature (described in Statistical analysis) were significantly different between the completed and discontinued groups at baseline (Table 1). However, the differences in sex, CGI-s scores and PANSS-General psychopathology scale scores between the two groups were marginally significant (*p* = 0.059, *p* = 0.123 and *p* = 0.090, respectively) at baseline (Table 1). Thus, baseline DAI-10 total scores, sex, CGI-s scores and PANSS-General psychopathology scale scores were included as covariates in the Cox regression model to determine predictors of discontinuation.

**Table 1:** Demographics of schizophrenic patients at baseline

	Completed Group		Discontinued group	Statistics			
	(*n* = 96)		(*n* = 104)	(Z /*X*²)		*P* value	
Sex (M/F)	39/53		55/43	3.579		0.059	
Age (years) (Mean ± SD)	25.41 ± 7.283		26.42 ± 8.088	-0.825		0.410	
Education (years) (Mean ± SD)	12.95 ± 2.500		12.50 ± 3.037	-0.615		0.539	
CGI-S (Mean ± SD)	5.19 ± 1.251		5.50 ± 0.765	-1.544		0.123	
PANSS-Positive (Mean ± SD)	23.22 ± 4.902		23.80 ± 6.282	-0.373		0.709	
PANSS-Negative (Mean ± SD)	20.56 ± 7.089		20.51 ± 7.447	-0.446		0.656	
PANSS-General psychopathology (Mean ± SD)	42.13 ± 7.417		40.46 ± 9.035	-1.695		0.090	
PANSS-G12 (insight)	5.48 ± 1.026		5.51 ± 1.123	-0.420		0.675	
PANSS total score (Mean ± SD)	84.77 ± 14.713		84.53 ± 16.255	-0.281		0.779	
Personal and Social Performance Scale (PSP)	40.01 ± 12.360		40.81 ± 15.545	-0.373		0.709	
Baseline DAI total scores	0.646 ± 5.039		-1.407 ± 4.964	-2.475		***0.013***	

Bold *p*-value indicates the result significant on the level of 0.05

**Fig. 1:**
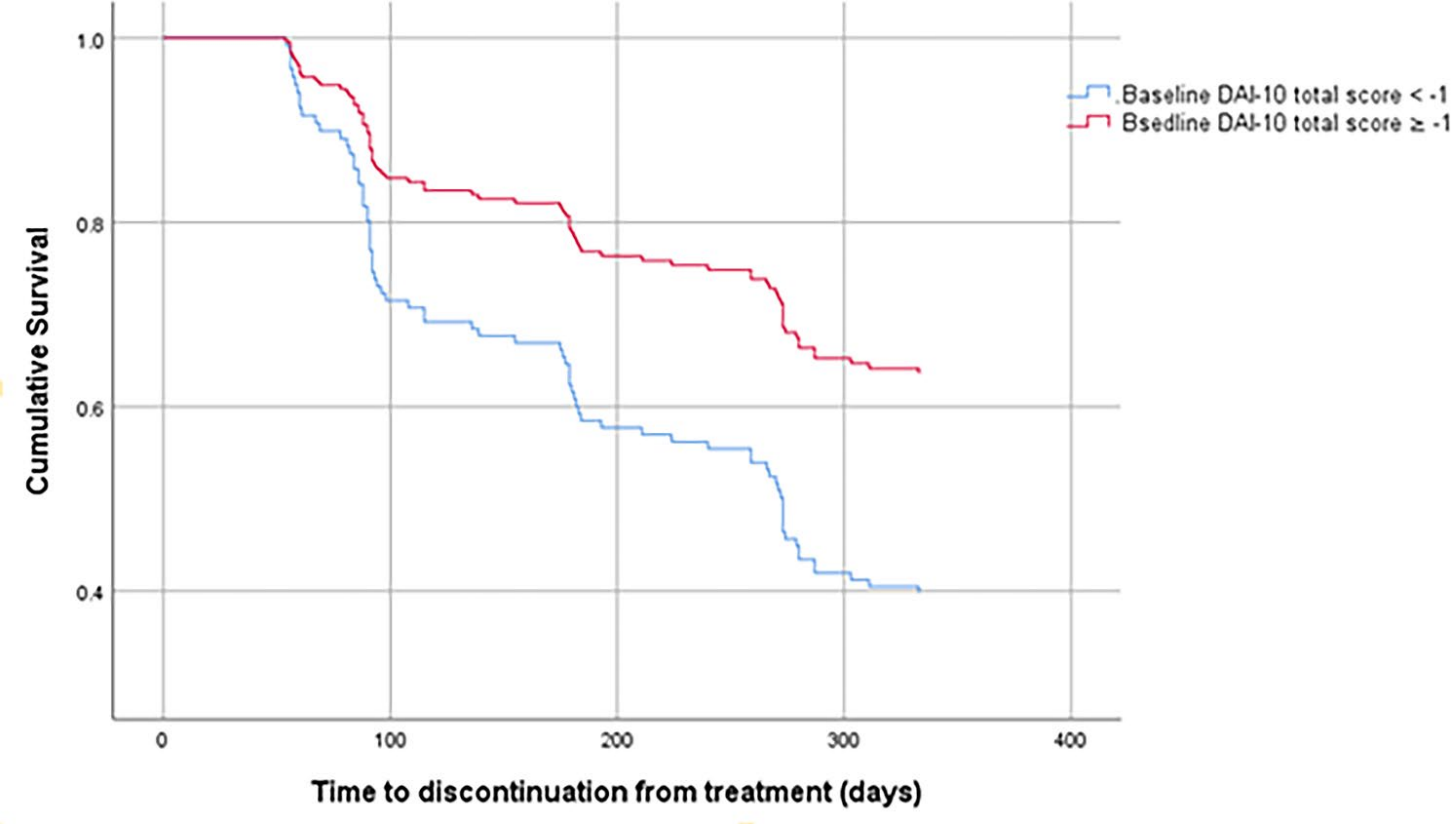
Survival curves of total 200 patients stratified by the cutoff point (-1) of baseline DAI-10. The red line was for the schizophrenic patients with baseline DAI-10≥-1. The blue line was for the schizophrenic patients with baseline DAI-10 <-1

**Fig. 2:**
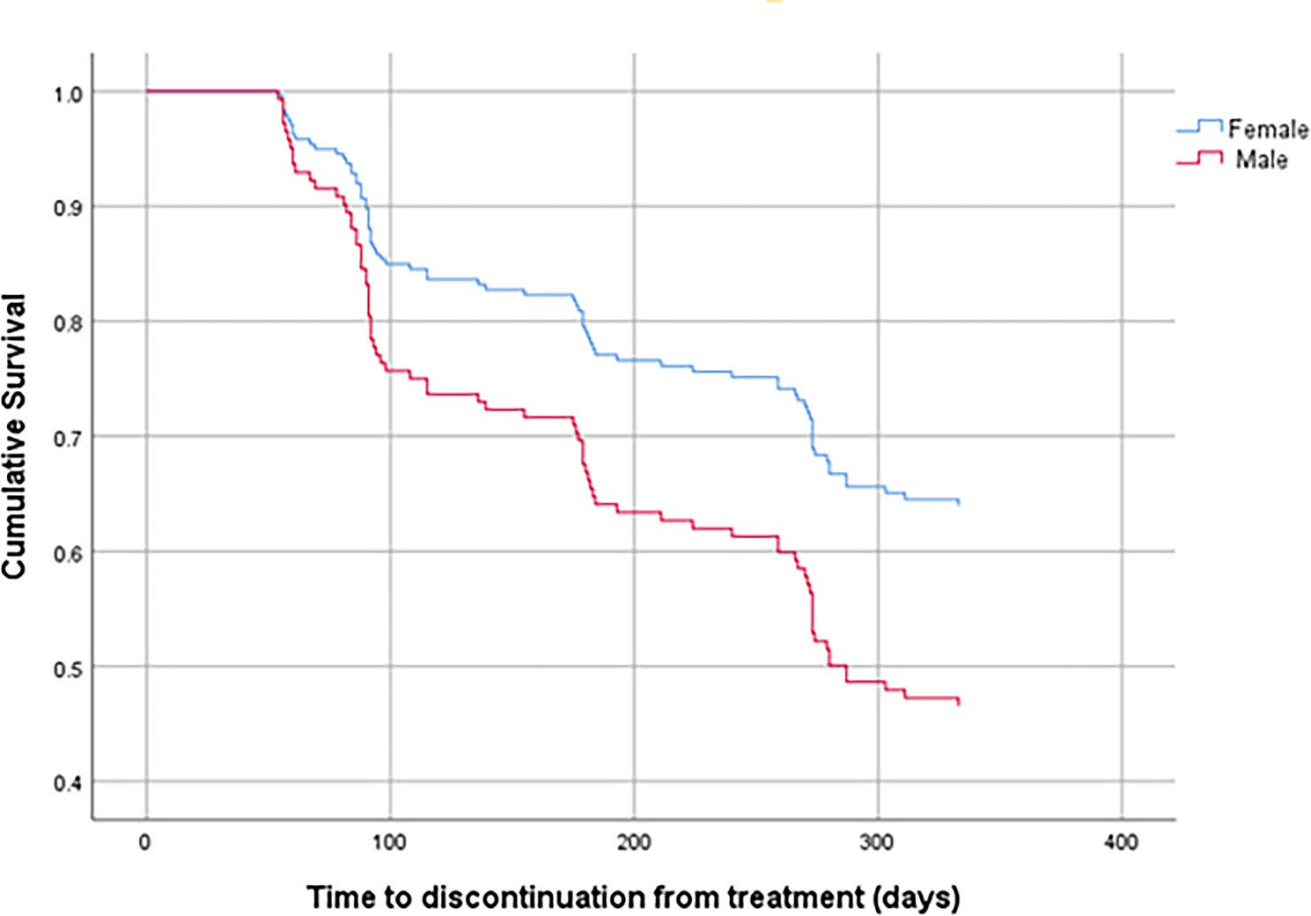
Survival curves of total 200 patients stratified by sex. The red line was for the male patients with schizophrenia. The blue line was for the female patients with schizophrenia

The Cox regression analysis revealed that male sex and lower DAI-10 total scores were independent risk factors for discontinuation (*p* = 0.030 and *p* = 0.006, respectively). The rate of discontinuation among patients with DAI-10 total scores <-1 was higher than that among patients with DAI-10 total scores ≥-1 (HR = 2.035, 95% CI: 1.290–3.211). The survival curves for baseline DAI-10 total scores and sex are shown in Figs. 1 and 2, respectively.

### Influencing factors of baseline DAI-10 scores

Multiple regression analysis revealed that CGI-s scores and PANSS-G12 (insight) scores were factors influencing baseline DAI-10 total scores (*p* = 0.005 and *p* = 0.030, respectively, Table 2). Higher CGI-s and PANSS-G12 scores were related to lower DAI-10 total scores at baseline, indicating that more severe symptoms and insight into the illness were associated with worse attitudes toward medication among patients with schizophrenia before treatment.

**Table 2:** Multiple regression model for baseline DAI-10 scores

*Vavirable*	β	*t*	*P value*	95%CI	
Sex	0.070	0.895	0.372	-0.861	2.285
Age (years)	-0.081	-1.052	0.294	-0.156	0.047
Education (years)	0.002	0.030	0.976	-0.279	0.288
CGI-S	-0.244	-2.838	**0.005**	-2.105	-0.377
PANSS-Positive	0.028	0.271	0.787	-0.159	0.209
PANSS-Negative	-0.067	-0.621	0.535	-0.209	0.109
PANSS-General psychopathology	-0.101	-0.874	0.384	-0.227	0.088
PANSS-G12 (insight)	-0.195	-2.196	**0.030**	-1.744	-0.092
PANSS total score	0.206	1.331	0.185	-0.036	0.182
Personal and Social Performance Scale (PSP)	0.041	0.480	0.632	-0.048	0.079
Constant		1.795	0.075	-0.844	17.574

Bold *p-*value indicates the result significant on the level of 0.05

## Discussion

The present study included a long observation period (52 weeks) and a large sample of Chinese patients with schizophrenia. A total of 430 patients with first-episode schizophrenia were recruited from six hospitals in China and were administered antipsychotic drugs. Of the 430 patients with schizophrenia, 96 patients reported DAI-10 scores at all 7 time points investigated, while 104 patients discontinued the study during the 1-year follow-up. Attitudes toward antipsychotic drugs at the beginning of the treatment (even before the treatment) were an important predictor of discontinuation during the treatment. At baseline, female sex and a DAI-10 total score greater than − 1 were independent protective factors against discontinuation of antipsychotic drug treatments during the 1-year follow-up. At baseline, the severity of the disease (CGI-s) and insight were shown to have an influence on baseline DAI-10 total scores.

In the present study, subjective attitudes toward drug compliance changed during the one-year follow-up treatment; however, only the baseline attitude was shown to be associated with discontinuation. This finding is supported by previous studies [6, 11]. Both Brain, Allerby [11] and Gaebel, Riesbeck [6] found that the baseline DAI score predicted medication continuation in a one-year follow-up study. In particular, consistent with the present study, Gaebel, Riesbeck [6] also examined first-episode schizophrenia patients.

Herein, baseline DAI scores were evaluated before starting continuous antipsychotic treatment. However, these scores could still predict medication adherence. A possible explanation may be that attitudes toward drugs among patients with schizophrenia have been reported to be associated with multiple factors, not only the experience with medications. Possible factors associated with attitudes toward drugs among patients with schizophrenia included self-stigmatization [25], duration of treatment [7, 26], duration of illness [27, 28], PANSS scores [13, 28], insight regarding illness [29], and illness severity [28]. In this study, baseline CGI-s and PANSS-G12 (insight) scores were associated with baseline DAI-10 total scores, indicating that the characteristics of the disease may influence attitudes toward drugs. Notably, it was reported that stigmatizing attitudes toward people with mental illness and psychosis are much stronger in Chinese culture than in European or American cultures [30, 31]. The cultural influences may shape the psychological factors aggravating insight deficits in schizophrenia, contributing to the association of insight with attitudes toward drugs in Chinese patients with schizophrenia.

Another possible explanation may be that factors related to the effects or feelings of drug treatment do not influence drug compliance via attitudes toward drug compliance. This is supported by findings from Robles Garcia and Salazar Alvarado [32], who reported that side effects during antipsychotic drug treatment were not biased to either adherent or nonadherent patients. Kuroda, Sun [19] compared attitudes toward medication and related factors for patients with schizophrenia in Japan and China. Although Patients in Tokyo had the higher proportion of antipsychotics polypharmacy and more complicated drug regimens, they did not have more negative attitudes toward medication than patients in Beijing. In particular, Kuroda, Sun [19] found that the side effects of medication were not associated with attitudes toward medication in patients with schizophrenia in China. Attitudes toward drug compliance may be more closely related to previous symptoms or experience. Kako, Ito [14] found that DAI-30 scores were correlated with past awareness and attribution subscale scores but not correlated with current awareness and attribution subscale scores. These findings indicated that attitudes toward medications may derive from patients’ subjective interpretation of their previous medicated state, thus affecting whether they continue the drug treatment [8, 32]. Although our sample comprised first-episode schizophrenia patients, the patients could also have previous experience related to schizophrenia (including suffering mental diseases, receiving some treatment, visiting the healthcare system or related descriptions from others) that impacted their attitudes toward drugs.

Additionally, medication adherence consists of a series of interrelated steps, including patients, their health providers and healthcare systems [33]. Subjective attitudes toward drug compliance may also be influenced by health providers, demographic factors (i.e., age [27] and sex [34]) and cultural factors [35]. Notably, compared to only 40% patients with schizophrenia living with their families in the United States, over 90% of Chinese patients with schizophrenia did so [36], leading to the tightly knit interdependence between patients and families. Thus, family-related factors (such as family caregivers’ affiliate stigma, knowledge related to schizophrenia, and income) were also found to influence schizophrenia patients’ attitudes toward drugs and treatment [37]. These factors may have an impact on attitudes toward drugs before formal drug treatment and can be difficult to change during the following treatment, thus leading to adherence or nonadherence during the treatment. Our study was conducted in China, and the findings suggest that researchers should focus on initial attitudes toward drugs among patients with schizophrenia in Chinese community settings.

In the present study, the cutoff value was suggested to be -1 based on the ROC analysis. This cutoff value was lower than that reported by Brain, Allerby [11]. A possible reason for the discrepancy may be that the patients recruited by Brain, Allerby [11] were not first-episode patients, and they had a longer duration of illness. Duration of illness and previous experiences with medication treatment have been shown to be associated with attitudes toward drugs [7, 13], thus potentially explaining the discrepancy between the previous study and the present study.

Previous studies reported that male patients with schizophrenia had more negative attitudes toward antipsychotic drugs than females with schizophrenia in both Western [38] and Chinese samples [34]. Our study did not find that sex was associated with baseline attitudes toward drugs. However, the present study indicated that males with schizophrenia had a higher risk of treatment discontinuation than females. This may be because our samples comprised first-episode drug-naïve patients. Gender differences may influence discontinuation via factors related to the following treatments. For example, females were more likely to seek help from others and more likely to have stronger and longer-lasting relationships with their families [38]. Furthermore, females may focus more on internalized feelings, which may lead to benefits from psychoeducation during treatment [39]. Additionally, in a Chinese study, female outpatients with schizophrenia (taking antipsychotic medication continuously for at least one month) were found to be related better attitudes toward drugs [19]. The above evidence supports that gender differences may influence adherence during subsequent treatment. Comparatively, before drug treatment (at baseline), gender differences may not have an impact on attitudes toward drugs in the present study. The severity of symptoms and insight into the illness, on the other hand, have an impact on attitudes toward medication in schizophrenia before treatment.

Our study suggests that baseline attitudes toward antipsychotic medication should be further investigated in order to improve adherence among patients with schizophrenia in clinical settings. Baseline attitudes toward drugs can be measured easily using the DAI-10; individuals with DAI-10 total scores less than − 1 may be more likely to discontinue treatment. Additionally, male sex, more severe symptoms and insight into the illness are potential direct or indirect risk factors for treatment discontinuation. Interventions or psychoeducation for improving adherence to antipsychotic treatment should focus on patients with these risk factors.

One limitation of our study is that we did not examine additional sociodemographic variables (e.g., marital status, income, and living area). These factors may also influence attitudes toward drugs. Another limitation of our study was the lack of data on psychotherapy or psychoeducation interventions at baseline or during treatment in the present study. Previous studies have reported that these interventions may improve medication adherence of schizophrenia patients [40] and reduce transition to psychosis in early stages of schizophrenia [41].Thus, psychotherapy or psychoeducation interventions at baseline or during treatment may have potential effect upon attitudes toward drugs. These limitations will be noted and improved upon in future studies.

## Conclusions

Attitudes toward antipsychotic drugs at baseline were shown to play a crucial role in predicting treatment discontinuation. At baseline, schizophrenic patients with male sex and a DAI-10 total score less than − 1 may be more likely to discontinue during the follow-up treatment.

## Data Availability

The datasets used and/or analysed during the current study are available from the corresponding author on reasonable request.

## Abbreviations

DAI-10: Drug Attitude Inventory-10
ROC: receiver operating characteristic
CGI: Clinical Global Impression
CGI-S: Clinical Global Impression – Severity scale
DSM-IV-TR: the Diagnostic and Statistical Manual of Mental Disorders, Fourth Edition, Text Revision
SCID-I/P: the Structured Clinical Interview for the Diagnostic and Statistical Manual of Mental Disorders, Fourth Edition, Text Revision (DSM-IV-TR) Axis I Disorder, patient edition
PANSS: Positive and Negative Syndrome Scale
PSP: the Personal and Social Performance
